# Association of long non-coding RNA *MIAT* and *MALAT1* expression profiles in peripheral blood of coronary artery disease patients with previous cardiac events

**DOI:** 10.1590/1678-4685-GMB-2018-0185

**Published:** 2019-11-14

**Authors:** Eman A. Toraih, Aya El-Wazir, Saleh A. Alghamdi, Ayman S Alhazmi, Mohammad El-Wazir, Mohamed M. Abdel-Daim, Manal S. Fawzy

**Affiliations:** 1 Genetics Unit, Department of Histology and Cell Biology, Faculty of Medicine, Suez Canal University, Ismailia, Egypt.; 2 Center of Excellence of Molecular and Cellular Medicine, Suez Canal University, Ismailia, Egypt.; 3 Medical Genetics, Clinical Laboratory Department, College of Applied Medical Sciences, Taif University, Taif, Saudi Arabia.; 4 Department of Clinical Chemistry, College of Applied Medical Sciences, Taif University, Taif, Saudi Arabia.; 5 Department of Cardiology, Faculty of Medicine, Suez Canal University, Ismailia, Egypt.; 6 Department of Pharmacology, Faculty of Veterinary Medicine, Suez Canal University, Ismailia, Egypt.; 7 Department of Biochemistry, Faculty of Medicine, Northern Border University, Arar, Saudi Arabia.; 8 Department of Medical Biochemistry and Molecular Biology, Faculty of Medicine, Suez Canal University, Ismailia, Egypt.

**Keywords:** CAD, lncRNA, MALAT1, MIAT, Real-time qPCR

## Abstract

Long non-coding RNAs (lncRNAs) are implicated in various cellular and pathological processes. Two lncRNAs, myocardial infarction-associated transcript (*MIAT*) and metastasis-associated lung adenocarcinoma transcript 1 (*MALAT1*), may be involved in the pathogenesis of coronary artery disease (CAD). Here, we aimed to determine the relative circulating levels of *MIAT* and *MALAT1* in 110 stable CAD patients and 117 controls and to correlate their levels with the clinical and laboratory data. Peripheral blood expression levels were quantified by Real-Time qPCR. The median *MIAT* expression level in CAD patients was significantly 12-fold higher than controls (*p*<0.001). Otherwise, the median *MALAT1* expression level was comparable in patient and control groups. Both lncRNAs showed significantly higher relative expression levels in patients with positive history of previous cardiac ischemic events, and *MIAT* showed significantly higher expression in diabetic CAD patients. The area under the curve of *MIAT* (0.888 ± 0.02 with sensitivity 95.5% and specificity 72.7%), was significantly larger than that of *MALAT1* (0.601 ± 0.04 with sensitivity 50% and specificity 63.6%) for detecting the presence of significant CAD. The current findings suggest that lncRNA *MIAT* could have a diagnostic significance in CAD patients. *MALAT1* levels, however, are not sufficiently reliable to have much clinical use in our cases.

## Introduction

In the past years, the discovery of long non-coding RNAs (lncRNAs) has piqued global interest. These RNAs are non-coding sequences of more than 200 nucleotides that are able to regulate gene expression through affecting transcription, post-transcriptional events, as well as translation (Rinn and Chang, 2012; Huang and Zhang, 2014). In turn, lncRNAs can influence various aspects of cell biology and disease development (Li *et al.*, 2013; Boon *et al.*, 2016). Several studies have detected and characterized the expression of lncRNAs in cardiac diseases and identified several types that control hypertrophy, cardiomyocyte apoptosis and mitochondrial function (Archer *et al.*, 2015; Uchida and Dimmeler, 2015). One of the early identified myocardial infarct (MI)-related lncRNAs is the myocardial infarction-associated transcript (*MIAT*), encoded by the five exon *MIAT* gene on chromosome 22q12.1 (Ishii *et al.*, 2006). *In vitro* functional analysis has revealed an MI susceptibility variant in exon 5 of *MIAT,* associated with an increased transcriptional level of the gene (Ishii *et al.*, 2006), suggesting a possible role of *MIAT* in the pathogenesis of MI. In addition, *MIAT* was shown to be involved in vascular endothelial dysfunction (Yan *et al.*, 2015), which suggests a role in the pathogenesis of atherosclerosis.

Another lncRNA with a possible relation to the pathogenesis of coronary artery disease (CAD) is *MALAT1* (metastasis-associated lung adenocarcinoma transcript 1), coded by the *MALAT1* gene on chromosome 11q13.1.(Michalik *et al.*, 2014). In the vascular system, this endothelial-expressed lncRNA can regulate vessel growth and function (Michalik *et al.*, 2014) and was proven to have a role in the regulation of endothelial cell proliferation (Liu *et al.*, 2014), as well as in the promotion of skeletal muscle differentiation (Watts *et al.*, 2013). Interestingly, levels of *MALAT1* have been shown to rise considerably with hypoxia (Michalik *et al.*, 2014). This might again point to a possible role of *MALAT1* in CAD, in which there is local hypoxia in the ischemic regions.

There are still several gaps in the existing knowledge. While *MIAT* variants have already been linked to MI (Ishii *et al.*, 2006), only a few controversial studies are available to date on the expression level of *MIAT* in peripheral blood of CAD patients (Vausort *et al.*, 2014; Liao *et al.*, 2016). In addition, *MALAT1* does have a role in endothelial cell function, but whether it plays a causative role in CAD is unknown. This study aims to determine the expression level of *MIAT* and *MALAT1* in CAD patients and to correlate these levels with the clinical and laboratory data of the patients.

## Subjects and Methods

### Study participants and protocol

This observational, case-controlled study included 110 nonconsanguineous stable CAD patients and 117 age- and sex-matched controls. Patients were consecutively recruited from the Cardiology Department, Suez Canal University (SCU) Hospital, Egypt, during the period between October 2015 and January 2016. Diagnosis of CAD was based on detailed history taking, clinical examination, resting electrocardiography and echocardiography, and further confirmed by coronary angiography (≥ 70% stenosis of at least one major coronary vessel caused by atherosclerosis) (Fawzy *et al.*, 2017a, 2018). Patients with congenital heart disease, valvular heart disease or non-atherosclerotic CAD (e.g. vasculitis, fibromuscular dysplasia, etc.), were excluded. Controls had no past history of cardiovascular problems and had a normal resting ECG. Selective coronary angiography was not performed for controls following our institutional ethical guidelines that do not permit to apply invasive procedures for controls in research. The study was conducted in accordance with the guidelines in the Declaration of Helsinki and approved by the Medical Research Ethics Committee of Faculty of Medicine, Suez Canal University (approval no. 2714). All participants enrolled in this study provided a written informed consent.

As part of risk assessment, the body mass index (BMI) of all participants was calculated. Hypertension was defined as repeated blood pressure ≥ 140/90 mmHg or regular use of anti-hypertensive drugs. Diabetes was defined as receiving hypoglycemic drugs, blood sugar > 11.1 mmol/Lon admission, or fasting hyperglycemia ≥ 7 mmol/Lin two determinants. Dyslipidemia was defined as serum triglycerides (TG) ≥ 1.7 mmol/L, total cholesterol (TC) > 2.3 mmol/L or high-density lipoprotein-cholesterol (HDL-c) < 1.0 mmol/L in males and < 1.3 mmol/L in females, or the intake of lipid-lowering drugs (Jellinger *et al.*, 2017). Other risk factors, such as smoking and family history (FH) of premature CAD, first degree male relatives <55 years or females <65 years were documented. The Framingham Risk Score was used to estimate the 10-year cardiovascular risk in participants. Low risk was defined as a score less than 10%, intermediate risk between 10 and 20%, and high risk over 20% (Ford *et al.*, 2004). Thrombolysis In Myocardial Infarction (TIMI) score Risk Index (TRI) was used to provide mortality estimates in CAD patients in terms of mild, intermediate, and high risk (Antman *et al.*, 2000).

A two-dimensional conventional echocardiographic study was performed for all patients using the standard views to exclude the presence of structural heart disease using a commercially available system (General Electric Healthcare Company, Vivid seven Dimensions Vingmed and Horten- Norway) with the 2.5-MHz phased array probe. All echocardiographic data were interpreted by two independent experts in echocardiography.

Selective coronary angiography was performed for all patients from the right femoral approach using the standard modified Seldinger technique (Schussler, 2011). CAD extent and severity were assessed according to our hospital protocol using the Gensini score (Saha *et al.*, 2015). The score is calculated based on the vessel affected, the location of the lesion, and the degree of stenosisl. This was determined and interpreted by two independent angiographers who were blinded to the clinical data.

### Specimen collection and laboratory investigations

Six milliliters of fasting blood samples were withdrawn from the median cubital vein of participants under aseptic conditions in sodium citrate, plain, and EDTA-Na_2_vacutainers. The citrated and clotted blood tubes were centrifuged at 2500 rpm for 20 minutes. Citrated plasma was immediately used to measure the prothrombin time (PT) using an automated analyzer (Sysmex CA-1500, USA), and the serum was kept at -20 °C in aliquots until the time of laboratory assessment. Fasting blood glucose(FBG), serum TC, HDL-c and TG were determined by the enzymatic method using a Hitachi 912 automated chemistry analyzer (Roche Diagnostics Co, Mannheim, Germany). Serum low density lipoprotein-cholesterol (LDL-c) was calculated by Friedewald’s equation, as all participants had TG values less than 4.5 mmol/L (Friedewald *et al.*, 1972). The EDTA tubes were used for subsequent genetic analysis.

### RNA extraction and reverse transcription (RT)

The total RNA was isolated freshly from whole blood using ABIOpure^TM^ Total RNA (Alliance Bio, USA, Catalog no. M541RP50-B) following the manufacturer’s protocol. RNA concentration and purity at the absorbance ratio 260/280 nm were determined by NanoDrop ND-1000 spectrophotometer (NanoDrop Tech., Inc. Wilmington, USA) followed by agarose gel electrophoresis to check for RNA integrity. The range of the extracted RNA was 20-65 ng/μL and the total RNA concentration in every sample was checked to be the same for the subsequent reverse transcription (RT) step. High Capacity cDNA Reverse Transcription Kit (Applied Biosystems, P/N 4368814) was used. For each 20 μL RT reaction, a 10 μL RNA sample was combined with 10 μL of RT reaction mix containing 2 μL of 10x Buffer, 0.8 μL of 25x dNTP (100 mM), 2 μL of 10x random primers, 1 μL of MultiScribe Reverse Transcriptase, 1 μL of RNase inhibitor, and 3.2 μL of nuclease-free water. RT was carried out in T-Professional Basic, Biometra PCR System (Biometra, Göttingen, Germany) at 25 °C for 10 min, followed by 37 °C for 120 min, and finally 85 °C for 5 min followed by a final hold step at 4 °C (Toraih *et al.*, 2018). All reactions included two types of controls: non-template control (NTC) and no-reverse transcriptase control. The cDNA products were subjected immediately to quantification by real-time PCR without storage.

### Gene expression analysis by quantitative Real-Time Polymerase Chain Reaction

Expression of *MIAT*, *MALAT1* and *GAPDH* (as an endogenous control) were quantified using pre-designed TaqMan® assays (Applied Biosystems, ID Hs00402814_m1, Hs00273907_s1, and Hs402869, respectively) and Taqman® Universal PCR master mix II (Applied Biosystems, P/N 4440043). The detailed description of each assay is available at (http://www.thermofisher.com). The PCR reactions were carried out in accordance with the Minimum Information for Publication of Quantitative Real-Time PCR Experiments (MIQE) guidelines (Bustin *et al.*, 2009) in a final volume of 20 μL, including 1.33 μL RT product, 10 μL TaqMan^®^Universal PCR Master Mix, 1 μL TaqMan^®^ assays. All reactions included three types of control: two controls for RT reactions and NTC for real time PCR (that contains nuclease free water instead of cDNA), to confirm the absence of non-specific PCR product generation. The PCR assays were performed in a StepOne Real-Time PCR System (Applied Biosystems) as follows: 95 °C for 10 minu followed by 40 cycles of 92 °C for 15 s and 60 °C for 1 min (Fawzy *et al.*, 2017b). Ten randomly selected study control samples were re-evaluated for the expression of the three studied genes in separate runs to test the reproducibility of the qPCR which showed very close C_q_ value results and low standard deviations. Serial dilutions by 1/10 to 1/100,000 of cDNA from a pool of CAD patients and three RT-PCR controls were run to test the sensitivity of the studied long non-coding RNAs.

### Statistical analysis

Appropriate sample size and power calculations were carried out using Quanto software package version 1.2.4 (http://biostats.usc.edu/software). Chi-square, Fisher’s exact, Student’s *t*, and Mann-Whitney U tests were used when appropriate. Logistic regression was performed to adjust for smoking status, lipid profile, platelet count, and white blood cell parameters (as *MIAT* was reported to be positively associated with the percentage of lymphocytes and negatively associated with neutrophils and platelets) (Sun *et al.*, 2018). Receiver operating characteristic (ROC) curves were generated to obtain the best cutoff values of both lncRNAs for discriminating CAD patients from controls. A two-tailed *p*<0.05 was considered statistically significant. The fold change of lncRNAs expression was calculated using the Livak and Schmittgen (2001) method based on the quantitative cycle (Cq) values with the following equation: relative quantity = 2^-DD*Cq*^, where ΔΔ*C*_*q*_ = (*C*_*q*_ LncRNA – *C*_*q*_ GAPDH) _CAD_ - (*C*_*q*_ LncRNA – *C*_*q*_ GAPDH) _Controls_. Data were managed using the Statistical Package for the Social Sciences (SPSS) for Windows (version 20.0).

## Results

### Baseline characteristics of the study population

The demographic, basic laboratory, and cardiac assessment parameters of CAD patients and controls are shown in Table 1.

**Table 1:** Baseline characteristics of CAD patients and controls.

Characteristics	Controls (n=117)	Patients (n=110)	*p*-value	Adjusted
				OR (95% CI) ^$^
Age mean (year)	53.6 ± 7.1	53.9 ± 6.3	0.208	
Sex				
Males	63 (53.8)	67 (60.9)	0.283	*Reference*
Females	54 (46.2)	43 (39.1)		0.5 (0.35-1.17)
Premature CAD	-	70 (63.6)		
Obesity	29 (24.8)	37 (33.6)	0.142	1.3 (0.89-2.55)
Smoking	17 (14.5)	39 (35.5)	**<0.001**	**3.3 (1.7-6.32)**
FH CAD	46 (39.3)	54 (49.1)	0.138	1.5 (0.7-2.64)
Hypertension	27 (23.1)	49 (44.5)	**<0.001**	**2.8 (1.4-4.45)**
Diabetes	25 (21.4)	30 (27.3)	0.299	1.4 (0.71-2.25)
Lipid profile				
TC (mmol/L)	4.39 ± 0.67	5.65 ± 1.53	**<0.001**	
TG (mmol/L)	1.45 ± 0.16	2.10 ± 0.54	**< 0.001**	
LDL-c (mmol/L)	2.01 ± 0.31	3.79 ± 1.50	**< 0.001**	
HDL-c (mmol/L)	1.26 ± 0.24	0.95 ± 0.30	**0.018**	
No of risk factors				
≤3	90 (76.9)	35 (31.8)	**< 0.001**	*Reference*
>3	27 (23.1)	75 (68.2)		**7.1 (4.02-11.8)**
Lesion type				
100% Normal	-	21 (19.1)		
< 50% Lesion	-	15 (13.6)		
100% Occlusion	-	20 (18.2)		
Lesion site				
Single VD	-	53 (48.2)		
Two VD	-	11 (10.0)		
Three VD	-	25 (22.7)		
CAD severity				
Gensini score	-	16 (3-53.5)		
TIMI score	-	20 (15.5-24)		

Cardiac catheterization showed that nearly half the patients had at least a single vessel disease (defined as luminal narrowing of > 70%); almost all of which have affection of the left anterior descending artery, while 10% and 23% of patients showed a two and three-vessel disease respectively. About a fifth of the patients had no discernible obstructive disease in the coronary angiogram. Less than 10% of the patients were classified as intermediate/high TRI, which denotes an in-hospital mortality risk > 10%. Variables for which statistically significant differences were found between the groups were considered for logistic regression correction.

### Expression profile of *MIAT* and *MALAT1*

**Figure 1:**
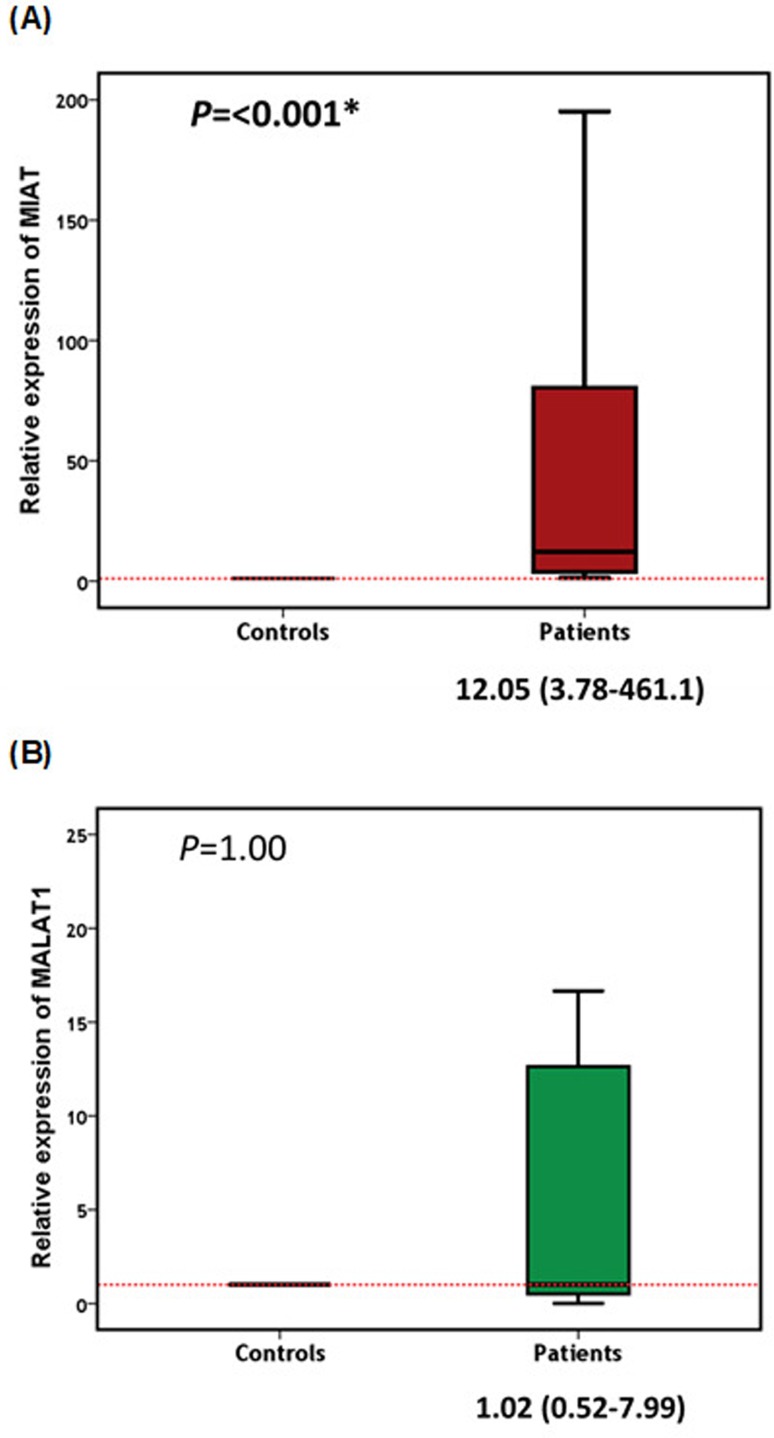
Peripheral blood expression levels of *MIAT* (A) and *MALAT1* (B) in patients and controls. Data are represented as medians. The box defines upper and lower quartiles (25% and 75%, respectively), and the error bars indicate upper and lower adjacent limits. Relative expression levels of *MIAT* and *MALAT1* were normalized to *GAPDH* expression level for each sample and calculated using the delta-delta Cq method [=2 ^(-DDCq)^] in comparison to controls. Mann-Whitney U test was used for comparison.

The endogenous control *GAPDH* was found uniformly expressed in all samples (26.9 ± 3.4 in controls, 27.924.58 in patients, *p*=0.229). In contrast, mean Cq values of *MIAT* and *MALAT1* varied significantly between the studied groups (*p*=0.005 and *p*=0.007, respectively). All samples exhibited up-regulation of *MIAT* with a relative expression ratio > 1.0. Nearly 27% of the patients (30/110) exhibited remarkably high levels, > 100-fold. The median *MIAT* relative expression level in patients was 12-fold (interquartile 457.3) higher than that in control individuals (*p*<0.001) (Figure 1A). Regarding the *MALAT1* expression profile, this was under-expressed (<1.0) in nearly half the patients, while the other half showed up-regulation. About 15% of the over-expressed samples was dramatically elevated by > 100-fold. The median (IQR) *MALAT1* level in patients was 1.02 (7.47), which was nearly similar to controls (Figure 1B).

**Figure 2:**
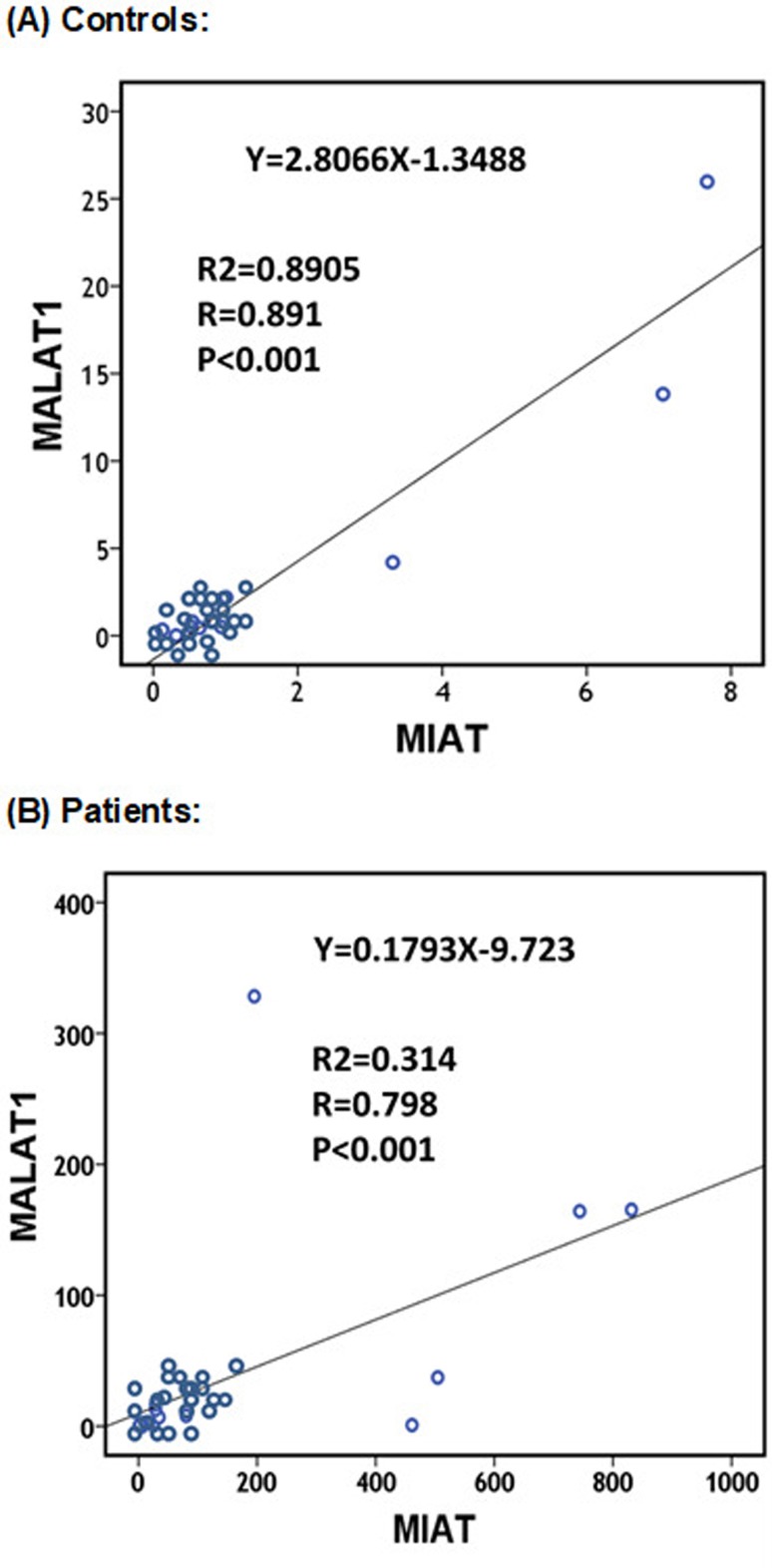
Correlation between peripheral blood *MIAT* and *MALAT1* in controls (A) and patients (B). Spearman’s rho binary correlation analysis was performed.

*MIAT* and *MALAT1*, being both pro-atherogenic lncRNAs (Jian *et al.*, 2016), showed a positive correlation in their expression profiles in both CAD patients and controls (r=0.798 and 0.891, respectively; *p*<0.001) (Figure 2). However, after removing the outliers, a direct correlation between *MIAT* and *MALAT1* expression levels still existed in the cases (r=0.620, *p*=0.008) but not in the control group (r=0.666, *p*=0.072).

### Association between the expression levels and clinical and laboratory features

*MALAT1* expression was significantly higher in patients with positive FH of CAD. When patients were classified into high and low expressers for circulating *MALAT1*, there were no differences in the baseline characteristics of CAD patients between the subgroups. Circulating *MIAT* was significantly higher expressed in diabetic CAD patients. Both *MIAT* and *MALAT1* expression levels were associated with hypertension and premature CAD (Table 2). Also, both lncRNAs showed higher relative expression in patients with a positive history of previous cardiac ischemic events. Furthermore, *MALAT1* showed higher expression in patients with previous history of stroke.

**Table 2:** Association between *MIAT* and *MALAT1* expression levels and clinical characteristics in patients.

Characteristics	Strata	No (n = 110)	MIAT	*p-*values	MALAT1	*p-*values
Age	<50 yr	35	34.7 (3.7-504)	0.139	1.1 (0.7-37.2)	0.105
	≥50 yr	75	6.8 (2.7-29.2)		2.7 (6.8-29.2)	
Sex	Female	43	18.7 (2.2-553)	0.737	10 (0.4-46.4)	0.545
	Male	67	12.1 (4.2-67.6)		1.2 (0.5-15.6)	
Risk factors	≤4 factors	80	6.8 (3.7-80.3)	0.828	0.9 (0.47-12.6)	0.652
	> 4 factors	40	21.2 (2.7-68.9)		1.12 (0.57-12.6)	
Obesity	Negative	73	46.1 (4.1-56.8)	0.404	0.9 (0.4-26.8)	0.312
	Positive	37	17.2 (3.1-31.9)		2.8 (0.81-9.7)	
Smoking	Negative	71	3.7 (2.5-8.2)	0.659	1.12 (0.5-12.4)	0.843
	Positive	39	26.0 (17.2-45.0)		4.8 (2.8-8.5)	
FH CAD	Negative	56	6.2 (3.7-28.6)	0.262	0.76 (0.47-1.68)	**0.002**
	Positive	54	29.2 (2.1-195.2)		6.8 (0.65-37.2)	
Hypertension	Negative	61	6.8 (3.7-68.9)	**0.042**	0.8 (0.51-7.9)	**0.035**
	Positive	49	17.2 (3.5-109.1)		1.4 (0.5-16.6)	
Diabetes	Negative	80	6.5 (3.7-80.3)	**0.008**	1.02 (0.51-13.6)	0.938
	Positive	30	25.2 (3.7-80.3)		1.3 (0.47-12.6)	
Dyslipidemia	Negative	49	6.2 (3.7-29.2)	0.913	0.7 (0.65-6.8)	0.445
	Positive	61	17.2 (3.7-80.3)		1.12 (0.51-12.6)	
Premature CAD	Negative	40	6.5 (3.0-27.7)	**0.009**	0.6 (0.49-12.9)	0.054
	Positive	70	23.2 (3.5-471)		1.9 (0.49-18.7)	
Previous event	Negative	20	3.2 (2.3-4.3)	**0.033**	0.3 (0.2-0.5)	**0.007**
	Positive	90	26.9 (5.8-166)		2.2 (06-15.6)	
Stroke	Negative	100	6.8 (3.7-34.7)	0.145	0.92 (0.48-11.4)	0.054
	Positive	10	137 (3.7-504.6)		22.6 (0.71-110.0)	
Lesion site	SVD	74	6.8 (3.7-195.2)	0.760	0.9 (0.51-12.6)	0.885
	MVD	36	25.2 (2.7-34.7)		1.6 (0.57-12.6)	
Occlusion	<50%	15	6.8 (2.7-34.7)	0.708	0.92 (0.47-12.6)	0.481
	≥50%	95	17.2 (3.7-461)		1.12 (0.76-7.99)	
Gensini score	Low	55	6.8 (3.7-80.3)	0.533	0.9 (0.51-10.3)	0.879
	High	55	25.2 (2.7-80.3)		1.12 (0.47-12.6)	
EF	≥ 50%	20	28.6 (3.7-80.3)	0.300	2.8 (0.51-10.3)	0.515
	< 50%	90	6.5 (2.7-34.7)		0.84 (0.51-12.6)	
Diastolic dysfunction	Mild	97	6.8 (3.2-34.8)	0.080	0.9 (0.51-10.31)	0.296
	Severe	13	54.8 (19.6-532)		4.5 (0.57-164.1)	

### ROC curve analysis

**Figure 3:**
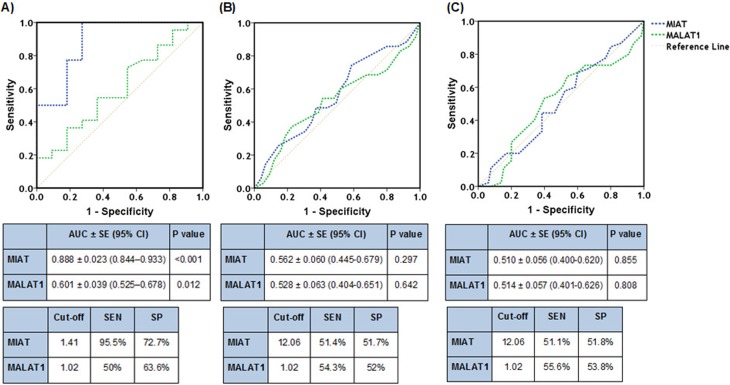
Receivers operating characteristic curves of lncRNAs *MIAT* and *MALAT1* using relative expression levels. Peripheral blood *MIAT* (blue line) and *MALAT1* (green line) expressions were plotted to determine their sensitivity and specificity at the cutoff fold change value for detecting the presence of significant coronary artery disease (A), multivessel disease (B), and high Gensini score > 20 (C) in CAD patients. AUC: Area under the curve; CI: Confidence interval; SE: Standard error; SEN: sensitivity; SP: specificity.

The AUC (area under curve) of *MIAT* was significantly larger than that of *MALAT1* for detecting the presence of significant CAD (Figure 3A). However, the AUC of *MIAT* and *MALAT1* were similar in predicting the presence of multivessel disease and high Gensini score (≥ 20) in CAD patients (Figure 3B, C).

## DISCUSSION

In the past decade, high-throughput sequencing analysis identified a group of lncRNAs that were found to be important regulators of gene function in many biological processes (Trapnell *et al.*, 2010). Disease-related deregulation of their expression was evident in various clinical entities, including cardiovascular disease (Zangrando *et al.*, 2014; Zhang *et al.*, 2016). More specifically, CAD has been characterized by a transcriptional reprogramming, which activates a network of cardiac signals that interact and converge on cardiac transcriptional factors. Collectively, these factors activate specific temporal and spatial programs to generate cardiac pathological remodeling controlled by lncRNAs (Archer *et al.*, 2015). Given that lncRNAs *MIAT* and *MALAT1* are known to affect endothelial function through alteration of specific signaling pathways that affect cellular proliferation, migration, and survival (Liu *et al.*, 2014; Yan *et al.*, 2015), we speculated on their potential role in the development of atherosclerosis and subsequent CAD.

In the present study, *MIAT* relative expression levels were elevated in peripheral blood of CAD patients, with a 12-fold higher relative expression level compared to healthy controls. In contrast, Vausort *et al.* (2014) found that *MIAT* was equally expressed in peripheral blood of both MI patients and healthy individuals, although they found a significant difference in its relative expression between ST elevation MI (STEMI) and Non-ST segment MI (NSTEMI) subgroups. While these results might seem contradictory to ours, we propose that this could be attributed to the time of *MIAT* relative expression quantification. The previous authors quantified *MIAT* expression levels within hours from the onset of the acute attack, while our assessments were made in already diagnosed stable CAD patients presenting for angiography, and not necessarily during the acute attack. This may suggest that *MIAT* levels are not initially raised during the acute attack, but rise later and remain elevated for an extended time. The significance of this, however, is yet unclear, but may point to a possible role of *MIAT* as an adaptive mechanism similar to the activation of the renin-angiotensin-aldosterone system that occurs in post MI patients (Sutton and Sharpe, 2000). Another finding in the current study that could further support this hypothesis is that patients with previous cardiac ischemic events have significantly higher levels of *MIAT* than those without.

Interestingly, it has been reported recently that *MIAT* functions as a miR-150-5p sponge that affects the repression of VEGF (vascular endothelial growth factor), which promotes inflammatory cellular migration into the intima, as well as inducing pathological angiogenesis (Zhu *et al.*, 2018). During the process of angiogenesis, which is a natural phenomenon after a CAD event, *MIAT* could significantly be up-regulated, subsequently sponging miR-150-5p, thereby up-regulating the level of VEGF and other factors contributing to atherosclerosis. Moreover, *MIAT* was recently found to be up-regulated in patients with ischemic stroke, a disease that shares a similar pathogenesis with CAD (Sun and Wong, 2016).

On the other hand, in the current study, circulatory *MALAT1* relative expression levels were up-regulated in half the patients undergoing coronary angiography. These relative expression levels were markedly elevated in patients with a history of previous ischemic events. This is consistent with the hypoxia-induced up-regulation of lncRNA *MALAT1* seen in ischemic limbs (Watts *et al.*, 2013). Recently, Sallé-Lefort *et al.* (2016) indicated that the enhanced *MALAT1* gene transcription upon low oxygen conditions was under the control of hypoxia-inducible factor 1α (HIF-1α), regulated by the activation of the CaMKK/AMPK (calcium/calmodulin-dependent protein kinase kinase/AMP-activated protein kinase) complex. At the cellular level, *MALAT1* was found to act as a decoy that binds to and interferes with the function of other RNAs or proteins (Tripathi *et al.*, 2010). In addition, it has been found to regulate genes that induce proliferation in endothelial cells, through enhancing cell cycle regulatory genes, particularly *CCNA2* (Cycline A2) gene, *CCNB1*, and *CCNB2*, and repressing the cell cycle inhibitor genes *p21* and *p27Kip1* (Michalik *et al.*, 2014).

In the current work, the *MALAT1* gene expression profile correlated strongly with *MIAT* expression levels in CAD patients, both in the control and CAD patient groups, supporting the shared significant roles these lncRNAs could play in vascular physiological or pathological processes (Jian *et al.*, 2016). Altered *MALAT1* expression was reported in previous studies and was closely related to various diseases, ranging from diabetic complications (Wu *et al.*, 2015), to a wide array of cancers (Ren *et al.*, 2016; Toraih *et al.*, 2018). Furthermore, *MALAT1* was one of the most up-regulated oxygen deprivation-responsive endothelial lncRNAs in ischemic stroke, implying a critical role in protecting the cerebral microvasculature from cerebral ischemic insults in mice (Zhang *et al.*, 2016).

Although correlation of *MIAT* and *MALAT1* levels with age was not statistically significant, it was found that their levels were significantly higher in premature CAD cases, being highly significant in case of *MIAT* and borderline in case of *MALAT1*. Circulating levels of *MIAT* and *MALAT1*, in addition, correlate with the severity of CAD using the gold standard, coronary angiography expressed in terms of the Gensini score, with *MIAT* being more sensitive in the detection of the presence of significant CAD. Another finding in the current study, was the link between positive family history for CAD and *MALAT1* levels, highlighting the presence of a genetic heritable factor and suggesting *MALAT1* as a putative risk factor for CAD. Moreover, the higher relative expression observed in CAD diabetic and hypertensive patient subgroups suggest a possible role of these types of lncRNA in the mechanisms by which diabetes and hypertension produce endothelial dysfunction and accelerate the rate of atherosclerosis (Dharmashankar and Widlansky, 2010; Kolluru *et al.*, 2012). Two recent studies reported the possible contribution of *MIAT* and *MALAT1* to endothelial dysfunction in diabetic patients (Puthanveetil *et al.*, 2015; Yan *et al.*, 2015). The first one showed that *MIAT* expression can be induced by hyperglycemia and is associated with the microvascular dysfunction occurring in diabetes. This could partially explain the high *MIAT* relative expression in our CAD diabetic patients versus the non-diabetic subgroup. The second study found an increase in endothelial *MALAT1* levels in response to hyperglycemia resulting in inflammation and subsequent endothelial cell dysfunction (Puthanveetil *et al.*, 2015).

## Conclusions

Collectively, whether lncRNAs *MIAT* and *MALAT1* have a causative role in CAD is still unknown, but our study confirms the possible association of their circulatory relative expression levels with coronary artery disease in our population. This association was more prominent in CAD patients with previous cardiac ischemic events. The current study was limited by the relatively small sample size and the fact that the samples were drawn only from outpatients presenting for coronary angiography, and not during the acute attack. We recommend further studies of a larger scale and serial measurements during the acute attack and at regular intervals afterwards. In addition, the molecular basis of the possible role of these lncRNAs as an adaptive mechanism should be further explored and established, as this might be an interesting target for future therapeutic interventions in post myocardial infarction patients.
